# Atranone-an underestimated secondary metabolite?

**DOI:** 10.1007/s12550-025-00609-x

**Published:** 2025-09-17

**Authors:** Mareike Dabisch-Ruthe, Jens Pfannebecker, Reinhard K. Straubinger, Frank Ebel, Sebastian Ulrich

**Affiliations:** 1 Microbiology, Department of Life Science Technologies, OWL University of Applied Sciences and Arts, Campusallee 12, Lemgo, 32657 Germany; 2 Chair of Bacteriology and Mycology, Department of Veterinary Sciences, Institute for Infectious Diseases and Zoonosis, Ludwig-Maximilians-University Munich, Sonnenstr. 24, Oberschleissheim, 85764 Germany

**Keywords:** Atranone, Secondary metabolite, *Stachybotrys*, Stachatranone, Stachybatranone

## Abstract

Atranones are secondary metabolites produced by *Stachybotrys chartarum*, a mold frequently found in water-damaged indoor environments. In contrast to the well-characterized and highly toxic macrocyclic trichothecenes, atranones have received relatively limited scientific attention. Approximately 60% of *S. chartarum* isolates from indoor environments produce atranones, while 40% form macrocyclic trichothecenes. No strain has been shown to produce both, indicating that the biosynthetic pathways for these two mycotoxin classes are mutually exclusive. Atranones are dolabellane-like diterpenoids synthesized from geranylgeranyl pyrophosphate through multiple enzymatic steps encoded by a specific core gene cluster. While the genetic structure of this cluster has been elucidated, its regulatory mechanisms remain poorly understood. Notably, although atranone-producing *S. chartarum* strains have been isolated from indoor settings, no study has yet confirmed the actual production of atranones in indoor environments, leaving the question of real-world exposure unresolved. Experimental studies in cell cultures and animal models indicate that atranones possess pro-inflammatory and cytotoxic properties, including the induction of apoptosis and cell cycle arrest. Atranone Q has demonstrated antitumor activity against osteosarcoma cells in vitro, and more recently identified derivatives such as stachatranone and stachybatranone have shown preliminary cardioprotective effects under ischemic conditions. However, these pharmacological effects remain exploratory and require further validation in in vivo models. Major knowledge gaps concern the environmental triggers for atranone biosynthesis, their regulation, actual presence in built environments, and potential health risks. These areas represent key priorities for future research.

## Introduction

Atranones are secondary metabolites produced by certain strains of *Stachybotrys* species, especially *S. chartarum* chemotype A and *S. chlorohalonata* (Andersen *et. al*. 2003). These molds are commonly found in water-damaged buildings worldwide and to some extent in straw (Hintikka 1977b; Andersen et al. 2002). While Research has focused on the highly toxic macrocyclic trichothecenes, atranones have received comparatively little attention despite being produced by 60% of all indoor isolated *S. chartarum* strains, while only 40% produced macrocyclic trichothecenes (Andersen et al. 2002). However, recent studies have shed new light on the biological relevance of atranones. Research has shown that they exhibit pro-inflammatory and cytotoxic effects in animal models and cell cultures, while individual atranone derivatives have even suggested therapeutic potential (Rand et al. 2006, 2011; Yang et al. 2019; Qin et al. 2020; Lin et al. 2024a, b). Despite this evidence, knowledge about atranones remains limited, with a manageable number of known compounds, only partial understanding of their biosynthesis, and a lack of systematic investigation into their effects on humans.

Our lack of knowledge raises the question of whether the potential of atranones may be underestimated due to their relative lack of representation in research compared to other known mycotoxins. The review aims to provide a comprehensive summary of the chemical, genetic, toxicological, and pharmacological properties of atranones and to evaluate their relevance based on the current research.

### Chemistry

Since the first isolation of an atranone from *S. chartarum* in 1999 (Hinkley et al. 1999), only 31 distinct atranones have been reported (Table 1), some of which showed antimicrobial activity, cytotoxicity, or enhanced neurite outgrowth capacities. Atranones Represent an uncommon type of C-alkylated dolabellane diterpenoid derivatives that are defined by a typical 5–11-fused bicyclic carbon skeleton (Hinkley et al. 1999, 2000). Atranones can be divided into three subsets: C_22_, C_23_, and C_24_ atranones. Among them, five compounds belong to the C_22_ atranones (atranones F − G, K, and O − P), two compounds are C_23_ atranones (atranone J, Q), and 24 compounds are C_24_ atranones (atranones A − E, H − I, and L − N, R-Z, methylatranones A − C, 22-epi-methylatranone B, and benzylatranone B) (Yang et al. 2019; Qin et al. 2020; Lin et al. 2024a).

**Table 1.**
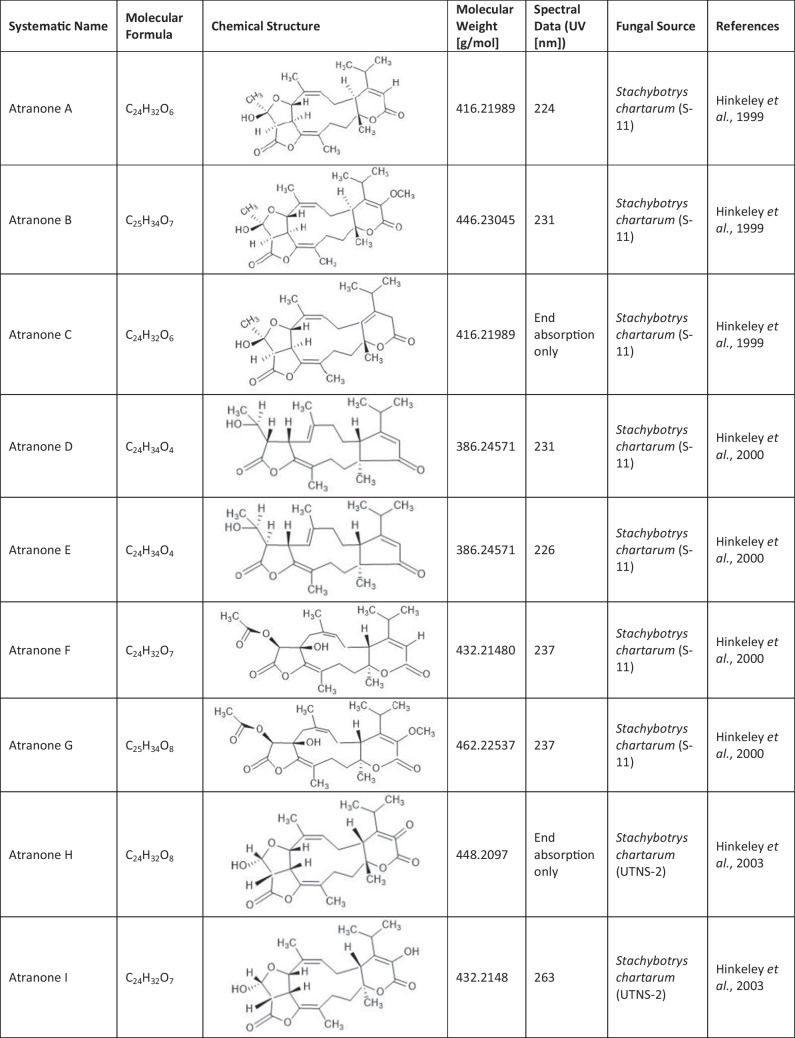

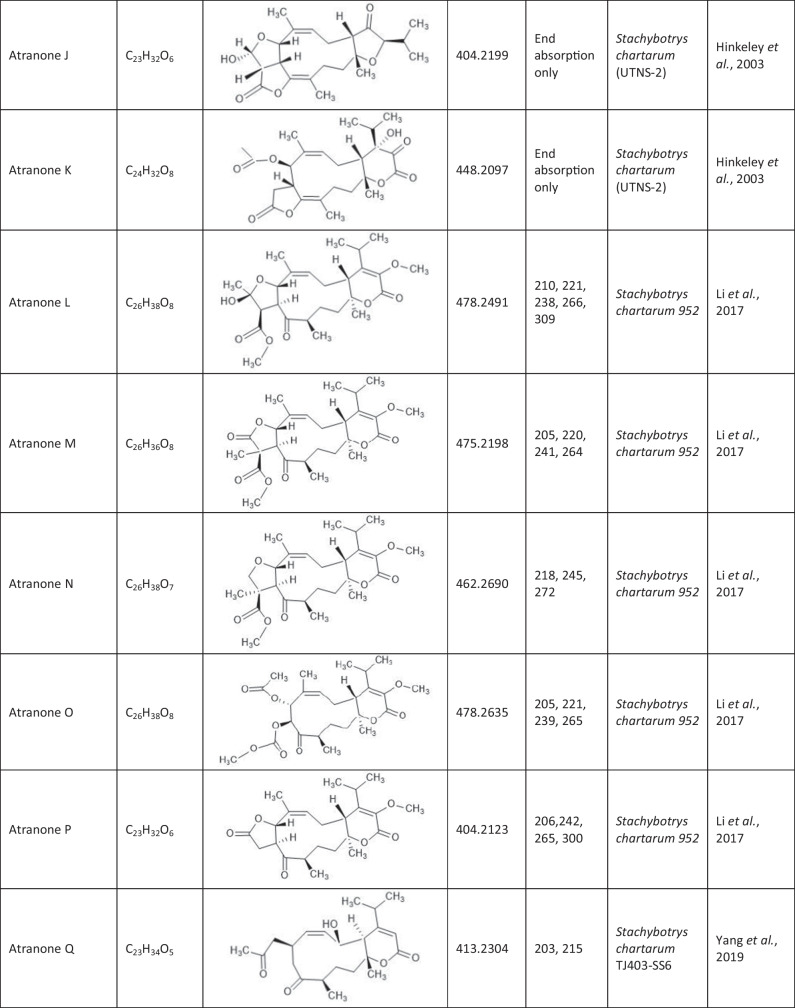

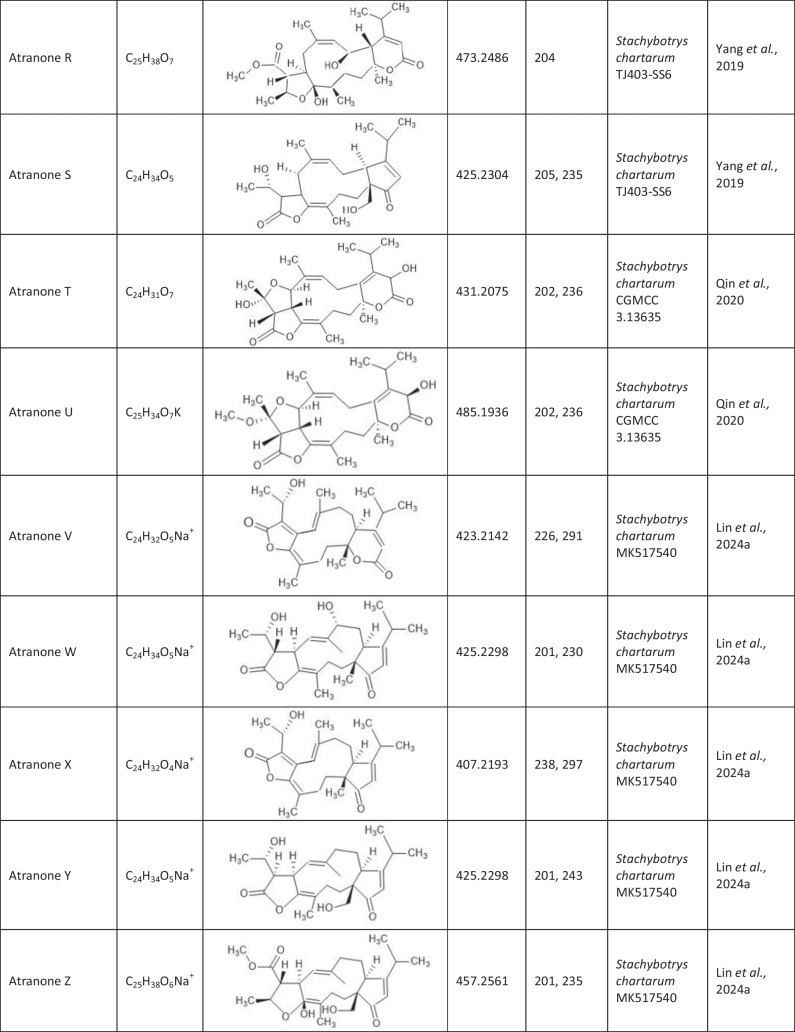

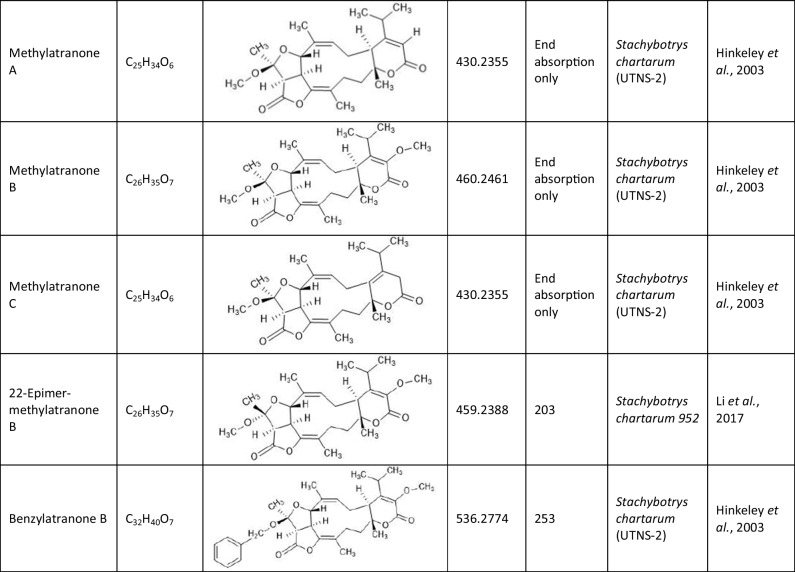
Compilation of all known atranones

**Fig. 1:**
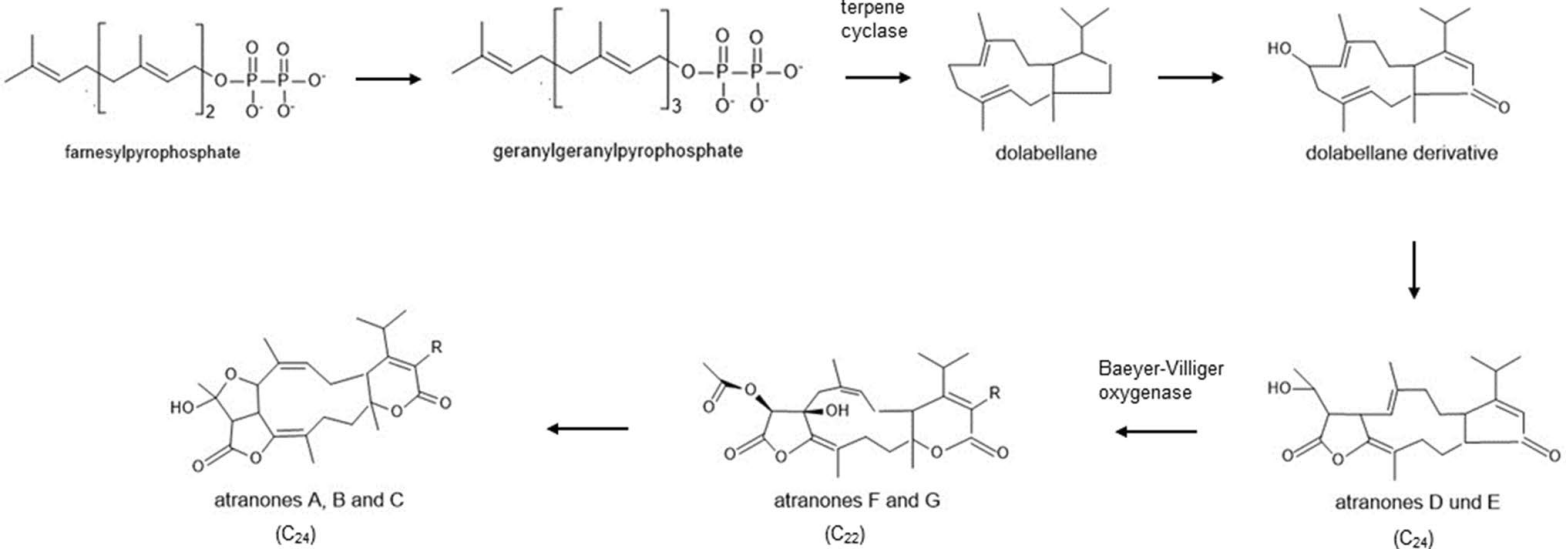
The biosynthesis of atranones. Figure was created based on data from Hinkley and Semeiks (Hinkley et al. 2000; Semeiks et al. 2014)

The synthesis of atranones is a complex, multi-step process that can be achieved both through natural biosynthesis and laboratory-based chemical synthesis. Detailed information on the chemical synthesis routes for all atranones that can be carried out in the laboratory is provided in the additional information part. Atranone biosynthesis is summarized in Fig. 1 (Hinkley et al. 1999, 2000, 2003; Hinkley and Jarvis 2000; Semeiks et al. 2014). The synthesis of atranones is based on isoprenoid precursors, such as farnesyl pyrophosphate (FPP), which are formed naturally through the mevalonate or methylerythritol phosphate pathway. Atranones arise from simpler diterpenes, the dolabellanes, which are synthesized through terpenoid pathways. These compounds are then converted into atranones through a series of enzymatic reactions. The conversion of FPP into atranones involves a specific sesquiterpene synthase (terpene cyclase) that catalyzes the formation of the atranone structure. Geranylgeranyl pyrophosphate (GGPP) is cyclized to a bicyclic compound, which is then converted into dolabellane. Subsequent enzymatic modifications, including oxidation, rearrangement, hydroxylation, and methylation (addition of methyl groups), lead to the formation of various atranone derivatives.

The specific enzymatic steps and intermediates involved in atranone synthesis can vary depending on the fungal strain and environmental conditions. For example, a Baeyer–Villiger oxidation converts atranones D and E to atranones F and G. Each atranone type can have different functional groups and arrangements depending on the specific enzymatic reactions.

### Genetics

#### Structural groups and chemotypes

*Stachybotrys* specific mycotoxins can be divided into three structural groups: macrocyclic trichothecenes (e.g., satratoxins), atranones, and phenylspirodrimanes (PSDs). Biosynthesis of atranones and trichothecenes shares the same “starting point”– FPP. Atranones are much less well studied, and little is known about their synthesis (Hinkley et al. 2000; Hinkley and Jarvis 2000). Andersen et al. characterized 122 *Stachybotrys* isolates from water-damaged buildings (Andersen et al. 2002). None of these isolates were able to produce both macrocyclic trichothecenes and atranones. Forty percent of these *S. chartarum* strains produced macrocyclic trichothecenes and 60% atranones (Andersen et al. 2002). To date, no strain has been described that produces neither nor both types of secondary metabolites. Whether the biosynthetic pathways of macrocyclic trichothecenes and atranones are indeed mutually exclusive or whether they can coexist in one fungus is an interesting question that has to be answered by genetic experiments.

#### Gene clusters and biosynthesis

Semeiks and colleagues performed a comparative genome sequencing of four *Stachybotrys* strains that belonged to different chemotypes characterized by the production of either atranones or satratoxins (Semeiks et al. 2014). This study revealed several chemotype-specific gene clusters that are predicted to encode enzymes for the synthesis of the respective secondary metabolites. This description of distinct genotypes, genotype A for atranone and S for satratoxin biosynthesis, led to a first genetic model for the biosynthesis of these mycotoxins by *S. chartarum.* The largest gene cluster encodes 14 proteins and was named the *core atranone cluster* (*cac*) or *atranone cluster 1* (*ac1*). All known atranone species derive from products of the *cac*, which was analyzed by Semeiks and colleagues. According to the Hinkley model of atranone biosynthesis (Hinkley et al. 2000), two characteristic reactions take place: the initial cyclization of GGPP to dolabellane and a Baeyer–Villiger oxidation that converts atranones D and E to atranones F and G. Two genes (*atr*13 and *atr*8) may be responsible for the initial cyclization and the Baeyer–Villiger oxidation, respectively.

How the atranone biosynthesis is regulated is still an open question, since no putative transcription factors or other regulatory genes have been identified within the *cac* or nearby. From the 122 *Stachybotrys* strains isolated from water-damaged buildings, all isolates belonging to genotype A produce simple dolabellane derivatives in culture, but not all of them produced atranones in the same experimental setting (Andersen et al. 2002). This led to the hypothesis that only some enzymes in the pathway are regulated at the level of transcription.

A second atranone-specific gene cluster, *ac2,* is smaller than *cac* and contains only six genes (Semeiks et al. 2014). In contrast to *cac*, the available *ac2* sequences lack neighboring genes on one flank and may therefore be incomplete. Evidence from this study suggested a potential role of phosphate sensing in the control of *ac2* activity, since phosphate-substituted compounds are required for the synthesis of terpenes. Hence, a specifically regulated phosphate transport may be necessary for the production of FPP or other atranone precursors. Semeiks and colleagues found that three genes of the *ac2* are homologous to those of a second locus that is conserved in all *S. chartarum* strains not only those that produce atranones. A more recent study showed that *ac2* occurs in the genomes of both, macrocyclic trichothecenes and atranone producers (Ulrich et al. 2022). In both genotypes, *ac2* is located at different positions, suggesting that these clusters were acquired in distinct events or that the respective regions have undergone significant genomic rearrangements. Accordingly, it is unlikely that *ac2*-encoded proteins are directly involved in the production of atranones.

The currently available knowledge on atranone biosynthesis is mainly based on two publications by Hinkley et al. (Hinkley and Jarvis 2000; Hinkley et al. 2003) and suggests GGPP to be the first intermediate that is most likely generated by ATR13, which is predicted to be a terpene cyclase. The putative functions of the different enzymes are summarized in Table 2. Apart from their function, it will also be interesting to identify their location within the cell, since evidence for other mycotoxins indicates that compartmentation is an important element in the organization of these biosynthetic pathways (Boenisch et al. 2017).

**Table 2:** Summary of the predicted CAC encoded ATR proteins of *S. chartarum* genotype A and their putative function according to Semeiks and colleagues (Semeiks et al. 2014)

ATR protein	Predicted function in *S. chartarum*	ATR protein	Predicted function in *S. chartarum*
ATR1	unknown	ATR8	Baeyer–Villiger monooxygenase
ATR2	Cytochrome P450 monooxygenase	ATR9	Short-chain dehydrogenase/reductase
ATR3	Cytochrome P450 monooxygenase	ATR10	Short-chain dehydrogenase/reductase
ATR4	Cytochrome P450 monooxygenase	ATR11	unknown
ATR5	Polyketide transferase	ATR12	O-methyltransferase
ATR6	Highly reducing polyketide synthase	ATR13	Terpene cyclase
ATR7	Short-chain dehydrogenase/reductase	ATR14	Cytochrome P450 monooxygenase

#### Genetic differentiation and evolution

The production of atranones and macrocyclic trichothecenes appears to be mutually exclusive and thereby defines the respective chemotypes (Andersen et al. 2003). Previous studies were unable to differentiate strains of the A and S chemotypes by cultural or morphological features (Andersen et al. 2003). Furthermore, various analytical techniques, such as matrix-assisted laser desorption/ionization time-of-flight mass spectrometry (MALDI-TOF MS), a loop-mediated isothermal amplification (LAMP) assay using neutral red for visual signal detection, and Fourier-transform infrared spectroscopy (FT-IR), have also failed to provide an unequivocal distinction between these two chemotypes (Gruenwald et al. 2015; Ulrich et al. 2016; Ekruth et al. 2020; Kock et al. 2021).

Independent of their mycotoxin profiles, a first differentiation of these strains was based on genetic information. Unlike in other fungi such as *Fusarium* and *Trichoderma*, the *tri5* gene of *Stachybotrys* is located within the *core trichothecene cluster* (*ctc*) (Semeiks et al. 2014), suggesting that *tri5* is part of the genetic program for trichothecene synthesis in this genus. It is furthermore assumed that, similar to *Trichoderma*, the product of Tri5 in *Stachybotrys* probably does not undergo hydroxylation at C-3, which contributes to a specific toxin biochemistry (Semeiks et al. 2014). Andersen and co-workers demonstrated that chemotype S strains could be differentiated from chemotype A strains by a C-to-T exchange in their *tri*5 genes (Andersen et al. 2003), which was later described as a silent mutation (Ulrich 2016). This means that all strains with a thymidine (T) at the relevant site within the *tri*5 gene belong to *S. chartarum* type A, produce atranones, harbor all *atr* genes (*atr*1-14) but lack all *sat* genes (*sat*1-21). Whereas all strains that produced macrocyclic trichothecenes (roridin E, -L2, verrucarin J, satratoxin G, H, and F) have a cytidine (C) at this position, belong to *S. chartarum* type S, harbor all *sat* genes but lack the *atr* genes. However, the type S-specific thymidine was also found in six *S. chartarum* strains that produced no macrocyclic trichothecenes (Ulrich et al. 2020). The latter finding indicates that the *tri*5 gene sequence is not a reliable marker for the identification of *S. chartarum* strains that produce macrocyclic trichothecenes.

**Fig. 2:**
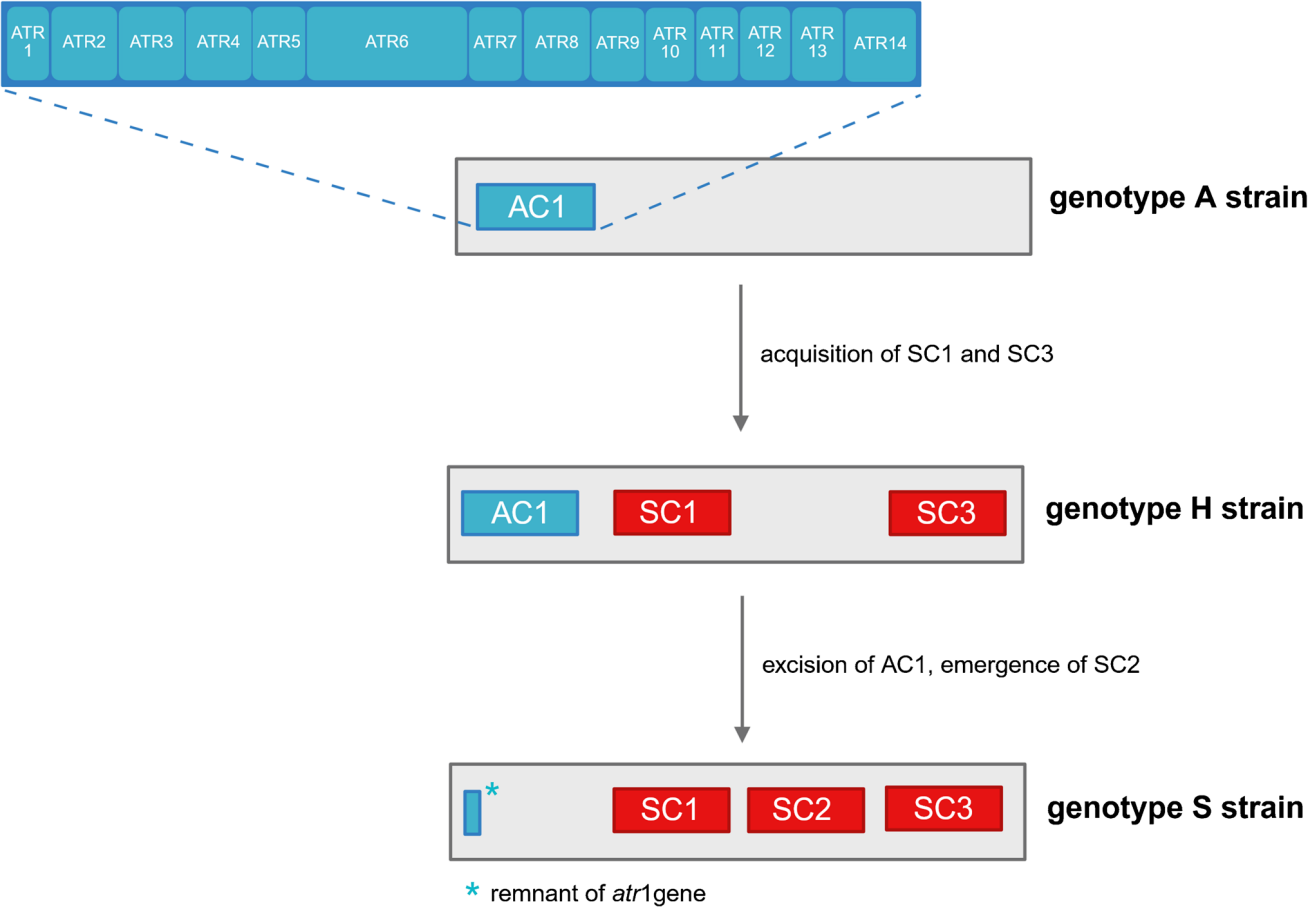
Current model of the evolution of the *S. chartarum* genotype A, H and S. The different gene clusters are depicted as boxes. The size and the position of these boxes do not represent the correct size and position of the respective cluster in the genome. Atranone gene clusters are shown in blue and satratoxin gene cluster in red. Figure was created based on data from Semeiks and Ulrich (Semeiks et al. 2014; Ulrich et al. 2022)

Because of these findings, a triplex PCR assay for chemotype differentiation was developed (Ulrich et al. 2020). PCR primers were designed to hybridize with the *sat19*, *atr6*, and *atr4* genes, respectively, to allow for specific identification. The *sat19* gene was selected because it seemed to be unique for type S strains of *S. chartarum* (Semeiks et al. 2014). The screening of 15 analyzed *S. chartarum* isolates revealed four strains with amplified DNA fragments typically found in type S strains, but also PCR products typical for type A strains (Ulrich et al. 2020). Based on these findings three *S. chartarum* genotypes were defined: genotype S (a single 346-bp fragment (*sat19*)), genotype A (a single 230-bp fragment (*atr6*)), and genotype H (a hybrid pattern with two fragments of 230 bp (*atr6*) and 346 bp (*sat19*)) (Ulrich et al. 2020). Further data revealed that the hybrid genotype H contains the complete gene set for atranone production but an incomplete set for the synthesis of macrocyclic trichothecenes and a first toxin analysis revealed no detectable macrocyclic trichothecenes (Fig. 2). The H-type strains possess all *atr*-, but only certain *sat*-genes. At first glance, the existence of the new genotype H in *S. chartarum* challenged the concept of mutual exclusiveness of the chemotype-specific gene clusters in *S. chartarum* (Semeiks et al. 2014). However, the incomplete *sat* gene cluster of these strains indicates that these genetic elements are tolerated, since they are presumably no longer functional. This observation is in agreement with studies by Peltola et al*.* (Peltolta et al. 2002) who developed a PCR assay based on the *tri5* gene in *S. chartarum* and found that 40% of the PCR-positive isolates did not produce any macrocyclic trichothecenes.

The existence of the genotype H raises the question of its genetic origin and its evolutionary relationship to the other genotypes. A first model for the evolution of the three *S. chartarum* genotypes was proposed after a deep re-sequencing of several strains combined with LC–MS/MS (Liquid chromatography–mass spectrometry) analysis of their mycotoxins (Ulrich et al. 2022). Figure 2 shows the possible relationship between the evolution of the individual genotypes. According to this model, genotype A represents the oldest form. The acquisition of *sc1* and *sc3* gene clusters led to genotype H. Genotype S thus arose from a loss of *ac1* and the concomitant acquisition of the *sc2* gene cluster. This leaves a genetic scar of *ac1* (Ulrich et al. 2022). This proposed evolutionary pathway still needs more data to be ratified.

Interestingly, atranone-producing strains lack either a complete *sc3* cluster (A-type) or at least the *sat21* gene (certain H-type strains) (Kock et al. 2021). Of the 105 *S. chartarum* strains mentioned above, none possessed or lacked both *ac1* and *sc2* (Kock et al. 2021) suggesting that these gene clusters are mutually exclusive. Thus, a switch to a certain strain from one genotype to the other requires the acquisition of one or more new gene cluster(s), which is only possible after complete removal of the original clusters. Strikingly, no strain representing the intermediate state, without *ac1* and *sc2,* has ever been identified. Although the exact pathway of macrocyclic trichothecenes or atranone biosynthesis is unknown, we know that FPP is the starting compound for both processes (Ueno 1983; Hinkley et al. 1999). Apparently, each strain has to “decide” at the genetic level whether to convert FPP into atranones or macrocyclic trichothecenes.

## Which organisms produce atranones?

Atranones have been found exclusively in species of the genus *Stachybotrys*, specifically in *S. chartarum* (Andersen et al. 2003), *S. chlorohalonata* (Andersen et al. 2003), and *S. microspora* (Andersen et al. 2002). The most well-studied and significant atranone-producing species to date is *S. chartarum*. At the sequence level, the *ac1* is well characterized and present in all A-type strains studied (Semeiks et al. 2014; Ulrich et al. 2020, 2022). *S. chlorohalonata* is closely related to *S. chartarum,* possesses an atranone gene cluster, and produces atranones, but has been less frequently studied (Andersen et al. 2003). The atranone gene clusters in *S. chartarum* and *S.* *chlorohalonata* show certain similarities, particularly with regard to the genes responsible for atranone biosynthesis. However, the exact organization and function of these clusters vary between species. Little is known about *S. microspora*, but atranone production has been documented in some isolates even though the gene clusters have not yet been studied in this species (Andersen et al. 2002; Abdeljalil et al. 2023). No other fungal genera have been identified as atranone producers, underscoring the genus-specific nature of this secondary metabolite and emphasizing the need for further research into its distribution and biological role.

## The importance of atranones as mycotoxins that affect humans and animals

As a hydrophilic fungus, *S. chartarum* is commonly found in humid buildings, contaminated indoor air, building materials, and wet animal feed and bedding material. Its potentially toxic mycotoxins, such as atranones or macrocyclic trichothecenes, are likely the reason why this fungus is associated with different types of disease in animals and humans. The most common symptoms in humans are fatigue, respiratory illnesses ranging from cough and mucous membrane irritation to bronchiectasis, alveolitis, and pulmonary fibrosis, as well as sick building syndrome, a multifactorial disease with nonspecific symptoms (Johanning and Yang 1994; Andersson et al. 1997; Engvall et al. 2001; Scheel et al. 2001; Usleber et al. 2001; Mussalo-Rauhamaa et al. 2010). Sick building syndrome is defined by symptoms of illness in the upper respiratory tract, headaches, fatigue, and skin rashes that are associated with people staying in a specific building and is not only linked to the presence of *Stachybotrys* spp. (Redlich et al. 1997; Fromme et al. 2016). Exposure to *S. chartarum* has also been linked to pulmonary hemorrhage in infants but is controversially discussed (CDC 2002; Dearborn et al. 2002; Miller et al. 2003) and in humid, water-damaged environments, the concentration of this fungus can easily exceed the sensitization threshold for allergy in susceptible individuals (Chung et al. 2010). Stachybotryotoxicosis is a severe disease in horses and the most important disease associated with *S. chartarum* in animals. The fungi preferentially grow as saprobiotics on moist, cellulose-containing, and wilting materials such as hay and straw, but also on cellulose-containing building materials and interior materials (Forgacs 1972; Hintikka 1977a; Hintikka and Nikulin 1998). The oral uptake of the toxins then causes mucosal necrosis or even death due to lung bleeding (Forgacs 1972). All the above-described pathological findings were always linked to satratoxins, and little is known whether they may be the consequence of combinatory effects or other metabolites.

While significant attention has been focused on the satratoxins, the role of atranones in human and animal health remains poorly understood. Recent studies, however, suggest that atranones may have various biological effects, including inflammatory responses and cytotoxicity, which raises concerns about their potential as emerging pathogenic factors (Flemming et al. 2004; Piecková et al. 2006; Rand et al. 2006, 2011; Miller et al. 2010; Rakkestad et al. 2010; Qin et al. 2020). Understanding the significance of atranones is crucial for assessing their impact on public and veterinary health, particularly in environments where mold contamination is a concern.

## Effects of atranones in animal models

In all studies published to date, spores of *S. chartarum* were taken up by mice through intratracheal instillation. The studies used different numbers of spores and different exposure times. Flemming and colleagues observed increased levels of pro-inflammatory cytokines such as interleukin-1 (IL-1), interleukin-6 (IL-6), and tumor necrosis factor-alpha (TNF-α) in the bronchoalveolar lavage fluid (BALF) of mice exposed to atranone-producing strains (e.g., JS 58–06) (Flemming et al. 2004). In a study with rats, chloroform-extractable metabolites of an atranone-producing *S. chartarum* strain caused significant lung damage after direct exposure to the respiratory tract (Piecková et al. 2006). An acute lung injury is indicated by a reduced viability of alveolar macrophages and an increased activity of cathepsin D. Treatment with purified atranone A and C showed significant inflammatory effects in mouse lungs, characterized by an increase in inflammatory cells and pro-inflammatory mediators (Rand et al. 2006). These inflammatory reactions can be subdivided into steps: First, an increased number of inflammatory cells, mainly macrophages and neutrophils, was observed 6–48 h after instillation, and this response was dose-dependent. Second, a significant increase in pro-inflammatory mediators was observed, including elevated concentrations of macrophage inflammatory protein (MIP-2), TNF-α, and IL-6. This response was particularly pronounced in animals that received higher doses of atranones (5–20 μg/animal), and occurred within a few hours after administration (Rand et al. 2006). The inflammatory response was both dose- and time-dependent, with more pronounced effects observed at higher doses. Treatment with atranone C influences gene expression in alveolar macrophages, leading to a significant response in inflammatory tissue. It was found that atranone C, in combination with other compounds, up- or down-regulated specific genes (MIP-2, TNF-α, nitric oxide synthase 2, Interferon λ) within the first two hours after exposure (Rand et al. 2011). Miller and his team were able to show that atranone C causes stronger pro-inflammatory responses than other fungal metabolites, such as brevianamide A, cladosporin, mycophenolic acid, neoechinulin A and B, sterigmatocystin, and TMC-120A (Miller et al. 2010).

Apart from inflammatory responses, atranones can also induce cytotoxic effects. In vitro studies have shown that the cytotoxicity of atranone-producing strains is generally lower than that of strains producing macrocyclic trichothecenes. Atranone-exposed mice showed significantly increased levels of BALF protein, albumin, IL-1β, IL-6, TNF-α, and lactate dehydrogenase, indicating an inflammatory response and cellular damage in the lung tissue (Flemming et al. 2004). Treatment with atranone C resulted in structural changes in lung tissue, such as thickening and epithelial detachment (Miller et al. 2010). However, in vivo studies have shown that exposure to *S. chartarum* spores, regardless of the chemotype (A or S), can cause significant lung damage and acute lethality (Flemming et al. 2004).

## Effects of atranones on human cells

Treatment of THP-1 cells, a human monocytic cell line, with spore crude extract of the atranones produced by *S. chartarum* isolate IBT 9634 (chemotype A) resulted primarily in necrotic cell death, whereas spore crude extract of the satratoxin-producing strain IBT 9631 (chemotype S) induced both apoptosis and necrosis (Rakkestad et al. 2010). These differences in the cellular response to the different spore extracts indicate the different toxic effects of the produced mycotoxins. However, it is not clear how these effects are also triggered or in part caused by other secondary metabolites such as PSDs, which are produced by both chemotypes.

Atranones furthermore exhibit significant cytotoxic effects against MG-63 human osteosarcoma cell lines (Qin et al. 2020). Purified atranone Q demonstrated a strong cytotoxicity, with an IC₅₀ value of 8.6 μM, outperforming 5-fluorouracil (IC₅₀ of 10.4 μM). Atranone Q induced apoptosis, as evidenced by chromatin condensation and nuclear fragmentation, caused a cell cycle arrest at G0/G1 phase, and thereby inhibited cell proliferation.

Apart from these two studies described above, there are no further studies investigating the effect of atranones on human cells. To date, the influence of atranones on human cells can only be deduced from studies with *Stachybotrys* spp., but it is not possible from these data to assess which secondary metabolites triggered the observed effects. Therefore, further studies are urgently needed to systematically investigate the effects of atranones on human cells.

## Are atranones toxic for humans and animals?

Studies in mice have shown that atranone C increases the expression of inflammation-associated genes in alveolar macrophages, which causes an increased recruitment of immune cells such as T cells and neutrophils (Rand et al. 2006, 2011). This inflammatory response can lead to acute and chronic respiratory disease, in particular if animals and humans live in a humid and mold-contaminated environment. Allergic reactions are a concern for both animals and humans and may also result from the inflammatory effects described above.

Apart from their inflammatory activities, some studies have shown that atranone A and C also exhibit specific immunotoxicological properties that can lead to different types of damage in different organs (Rand et al. 2006, 2011; Miller et al. 2010). This suggests that the compounds not only pose a risk to respiratory health but may also have a broader impact on the overall health of humans and animals.

While acute inflammatory reactions to atranones have been documented in animals, the long-term health effects of chronic exposure to these mycotoxins remain uncertain. These include not only the possibility of pneumonia or other respiratory diseases, but also systemic effects that could potentially have far-reaching consequences for the health of the host. The study by Rakkestad and his team highlights that the reaction to atranone and other mycotoxins can also lead to DNA damage, which in the long term may increase the risk of developing diseases, such as cancer (Rakkestad et al. 2010).

Although direct clinical data on atranone in humans are lacking, respiratory illness, irritation, and allergic reactions have been reported to be associated with indoor exposure to *S. chartarum*. The main concern associated with atranones areas are pro-inflammatory effects affecting the human respiratory tract, especially after prolonged or high-level exposure. However, further studies are needed to further define the exact effects and risk groups.

## Pharmacological significance of atranones

To date, only one study by Qin and colleagues can be consulted to assess the pharmacological significance of atranones (Qin et al. 2020). They demonstrated that atranone Q exhibits significant cytotoxic activity with an IC_50_ value of 8.6 µM, making it more effective than the commonly used chemotherapeutic agent 5-fluorouracil, which has an IC_50_ value of 10.4 μM (Qin et al. 2020). However, the difference between these values is relatively small, and it remains unclear whether it is statistically or clinically meaningful. As such, caution is warranted in interpreting this difference as a strong indication of superior efficacy. Current osteosarcoma therapies primarily consist of a combination of surgical resection and chemotherapy. Atranone Q showed a significant increase in apoptosis rates from approximately 3.46% in control cells to 73.6% at the highest atranone Q concentration. These results demonstrate that atranone Q induces apoptosis and causes cell cycle arrest, particularly in the G0/G1 phase. This targeted mechanism of action could offer advantages in overcoming treatment resistance to conventional chemotherapies. Osteosarcomas frequently develop resistance to typical chemotherapeutic agents, which can lead to treatment failure and poor patient outcomes (Sellers et al. 2025; Zhra et al. 2025).

It is essential to stress that these observations are based solely on in vitro experiments. No in vivo or clinical data are currently available to confirm the efficacy or safety of atranone Q in living organisms.

In summary, while atranone Q shows preliminary promise as a potential antitumor agent by inducing apoptosis and disrupting the cell cycle in osteosarcoma models, its pharmacological relevance remains to be validated in further in vivo studies and clinical trials. These early findings highlight the need for continued research to determine whether atranones can contribute to the development of novel cancer therapies.

## Atranone derivatives: comparison of structure, toxicity and therapeutic potential

In addition to the atranones, similar components such as stachatranones (Yang et al. 2019; Lin et al. 2024a) and stachybatranones (Lin et al. 2024b) have been isolated in recent years from a marine-derived *S. chartarum*, but they differ in their chemical structures and their biological activities: Atranones typically have a tricyclic structure known as the atranone skeleton, which can include specific functional groups that vary among different atranone derivatives. In contrast, stachatranones are categorized as dolabellane-type diterpenoids indicating that their structure includes a specific diterpenoid framework. They are characterized by distinct cyclic arrangements and functional groups that differentiate them from atranones (Yang et al. 2019; Lin et al. 2024a). Stachybatranones specifically refer to a novel set of natural products (designated as stachybatranones A–F) isolated from *S. chartarum*. All new components are listed in Table 3 which also comprises their molecular formula and the fungal source. These compounds have unique structural features, including an 11-membered carbocyclic system and a five-membered hemiketal Ring, which distinguishes them from other atranones. For example, stachybatranone A Represents the first example of a 5–11-6-fused atranone featuring a 2,3-butanediol moiety (Lin et al. 2024b).

**Table 3.**
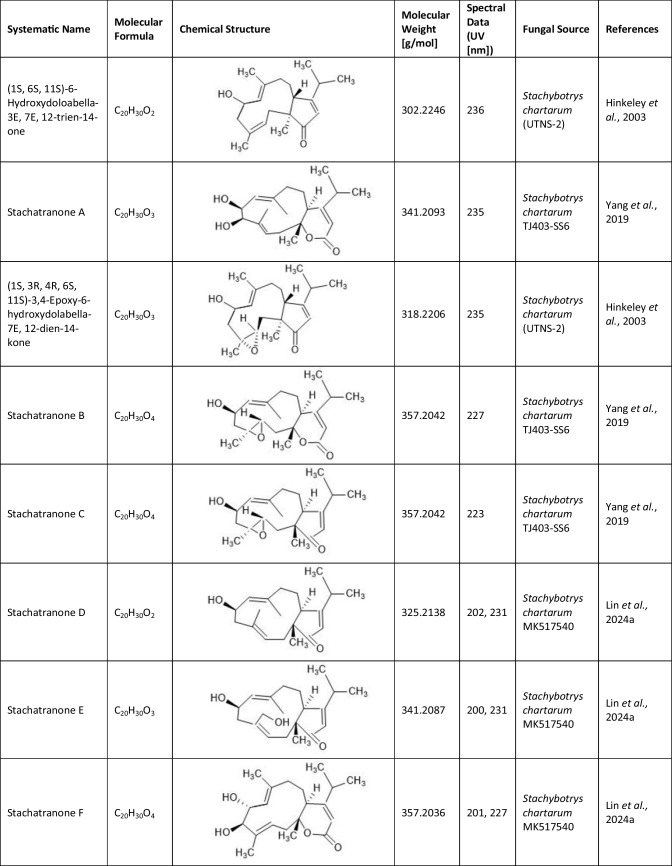

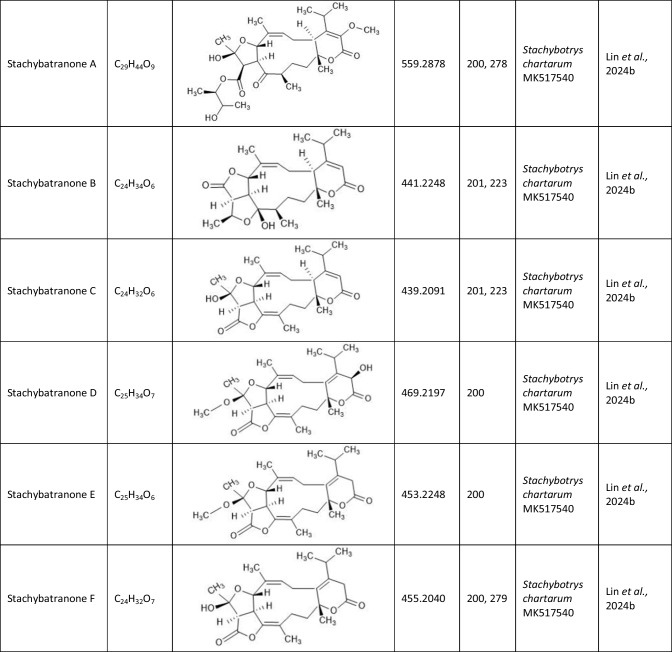
Compilation of all known stachatranone and stachybatranone

Lin and colleagues found that stachatranone and stachybatranones protect cardiomyocytes against cold ischemic injury (Lin et al. 2024a, b). Stachybatranone A and D and stachatranone F, in particular, showed a dose-dependent effect, suggesting that their efficacy increases with concentration. Stachybatranone A and stachatranone F inhibited the dephosphorylation of the key signaling proteins PI3K and AKT, which are associated with cell survival and growth. This mechanism suggests that stachatranone and stachybatranone contribute to maintaining the function and viability of cardiomyocytes under ischemic conditions, and could thus represent a potential therapeutic option for improving the outcomes of heart transplants.

Cold-induced ischemic injury is associated with increased production of reactive oxygen species (ROS) resulting in oxidative stress and damage to cardiac cells. Stachatranone F possesses the ability to reduce ROS concentrations during cold ischemia, thus protecting against oxidative stress (Lin et al. 2024a).

In summary, stachybatranone A and stachatranone F act by preventing cell death through modulation of signaling pathways and by reducing oxidative stress. The identification of stachybatranone and stachatranone as cardiomyocyte protectants opens a promising approach to improve the outcome of heart transplantations, address ischemic challenges, and ultimately support patients with end-stage heart failure.

In conclusion, while atranone, stachatranone, and stachybatranone belong to the same category of compounds, they differ in their specific molecular structure, biological activities, and potential medical applications. Although little is known about the anti-inflammatory effect of atranone, initial results suggest promising possibilities for future medical applications.

## Conclusions

In contrast to the intensively studied macrocyclic trichothecenes, atranones – despite preliminary evidence of their pro-inflammatory, cytotoxic, and potentially pharmacologically relevant properties – have so far received only marginal scientific attention. The chemical and genetic complexity of atranone biosynthesis, the exclusivity of its formation in certain *Stachybotrys* species, and a still unclear regulatory control highlight the significant gaps in knowledge in this field of research. Moreover, the mutual exclusivity of atranone and macrocyclic trichothecenes production is an exciting model for investigating the genetic control of secondary metabolites. In addition to their still poorly defined role in inflammatory processes and cellular destruction in lung tissue, recent studies also reveal a previously little-explored pharmacological potential, particularly in the field of cancer research. However, it is important to note that, to date, no study has conclusively detected atranones in indoor environments. While *Stachybotrys* strains with the genetic potential to produce atranones have been isolated from such settings, the actual production and environmental presence of these metabolites remain unproven. This raises a key open question: are atranones truly produced under indoor environmental conditions, or is their biosynthesis limited to specific, laboratory settings? Epidemiological evidence and risk assessment also remain largely lacking. While experimental studies in animals and cell culture models have demonstrated toxicological effects of atranones—particularly inflammation and cytotoxicity—there is a striking absence of epidemiological data linking atranone exposure to specific health outcomes in humans. Furthermore, no dose–response relationships or exposure thresholds have been established for atranones, and risk assessments are currently limited by insufficient data on environmental concentrations, human exposure routes, and susceptible populations. To adequately assess their potential impact on public health, systematic epidemiological investigations and environmental exposure studies are urgently needed.

## Isolation/Purification of atranones

### Atranones A-G

After four weeks of growth, a rice culture of *S. chartarum* chemotype A was extracted with methanol-chloroform and, following solvent Removal, the crude extract triturated with hexane to give 11.50 g of black gum. The extract was then separated chromatographically through polyethyleneimine (PEI) silica columns, eluted with increasing proportions of methylene chloride in hexane, then increasing amounts of methanol in methylene chloride to give four fractions. Fraction 1 (eluted with hexane to 80% methylene chloride-hexane) was applied to a 2 mm Chromatotron plate and eluted with 10% ethyl acetate in methylene chloride to yield atranones D and E. Fraction 2 (eluted with 100% methylene chloride) was applied to a 2 mm Chromatotron plate and eluted with acetate-hexane-methanol (40:60:5) to give atranone B. A fraction that was eluted later from the plate contained a 2:1 mixture of atranones A and C. These were resolved by semipreparative high-performance liquid chromatography (HPLC). Fraction 3 (eluted with 1–20% methanol-methylene chloride) was loaded onto a 2 mm Chromatotron plate and eluted with ethyl acetate-hexane-methanol to give atranones F and G (Hinkley et al. 1999; Hinkley and Jarvis 2000).

### Atranones H–K

After four weeks of growth, a rice culture of *S. chartarum* chemotype A was spread on paper and dried overnight to give 384 g of dry material. The material was grounded, MeOH–CHCl_3_ (1:1) was added and the mixture was left overnight after 1 h of ultrasound agitation. The mixture was filtered and the material re-extracted twice with MeOH-CHCl_3_ (1:1). The combined solutions were concentrated resulting in a crude extract of brown gum suspended in a reddish oil. This material was adsorbed on PEI silica gel and the free-flowing powder applied to a PEI column. Elution with increasing proportions of CH_2_Cl_2_ in hexane, then increasing amounts of methanol (MeOH) in CH_2_Cl_2_, gave eight fractions. Fractions 4 and 5 (eluted with 60–80% CH_2_Cl_2_-hexane) were combined and adsorbed on silica gel, then applied to a silica gel column eluting with increasing proportions of ethyl acetate (EtOAc) in hexane. Test-tube fractions were combined based on thin-layer chromatography (TLC) analysis and atranone I and atranone J recovered. One fraction from the silica gel column was further purified using radial chromatography on a 2 mm plate (hexane to 1:1 ethyl acetate–hexane eluent) and yielded atranone H and atranone K (Hinkley et al. 2003).

### Atranones L, M and N and 22-Epimer-methylatranone B

The marine crinoid-derived toxigenic fungus *S. chartarum *952 was cultivated on rice culture medium containing 70g rice and 3% artificial sea water for 35 days at 25 °C. The whole fermented cultures were soaked with MeOH three times. Then the crude extracts were dispersed with water and extracted with hexane, chloroform and EtOAc. The chloroform extract was subjected to a column chromatography on silica gel eluting with PE-EtOAc of increasing polarity to obtain five fractions (A-E). Fraction C was fractionated by Sephadex LH-20 eluting with CH_2_Cl_2_:CH_3_OH (1:1) and then further purified by preparative HPLC to obtain atranone M and N. Fraction D and E were repurified using HPLC to obtain atranone L and 22-Epimer-methylatranone B (Li et al. 2017).

### Atranones O and P

The strain *S. chartarum* 952 was cultivated in potato dextrose broth culture medium on a Rotary shaker at 120rpm for 7 days at 25 °C. Then the fungal strain was statically fermented in 80 g soybean and 3% artificial seawater for 35 days. The whole fermented cultures were soaked with MeOH three times. Then the crude extracts were dispersed with water and extracted with hexane, chloroform and EtOAc. The chloroform fraction was evaporated under reduced pressure to obtain a brown–red oil residue, which was subjected to a column chromatography on silica gel eluting with different solvents of increasing polarity from petroleum ether to MeOH to yield three fractions (A-C). Fraction A was further purified using silica gel with CHCl_3_-MeOH gradient to obtain atranone O as yellow oil and three sub-fractions (Fractions A2-A4). Fraction A4 was further purified using Sephadex LH-20 to obtain atranone P (Li et al. 2017).

### Atranones Q, R and S and Stachatranone A, B and C

The *S. chartarum* strain TJ403-SS6 was cultivated on potato dextrose agar plates at 28 °C for 5 days to prepare seed cultures. Fermentation was continued after inoculation with 200 g of cooked Rice at 28°C for 28 days. Afterward, the solid material was extracted with 95% aqueous EtOH at room temperature and concentrated under reduced pressures to yield a combined residue, which was partitioned between EtOAc and H_2_O. The EtOAc extract was subjected to RP-C_18_ column chromatography eluting with MeOH − H_2_O to give seven fractions (A − G).

Fraction B was passed through a Sephadex LH-20 column with successive elution with CH_2_Cl_2_ − MeOH (1:1), which was further purified by semipreparative HPLC to give stachatranone B. Fraction C was separated into three main fractions (C1 − C3) with a Sephadex LH-20 column. Fraction C2 was chromatographed on silica gel column chromatography using gradient elution with CH_2_Cl_2_ − MeOH to yield eight fractions (C2-1 − C2-8). Fraction C2-2 was repeatedly purified by semipreparative HPLC to provide stachatranone A, stachatranone C, atranone Q, and atranone S. Fraction C2-3 was applied to a silica gel column using petroleum ether − acetone and followed by semipreparative HPLC to obtain atranone R (Yang et al. 2019).

### Atranones T and U

Spores of *S. chartarum* CGMCC 3.13635 were directly inoculated in fermentation media (consisted of glucose (1.0%), peptone from porcine meat (0.5%), yeast powder (0.5%), KH_2_PO_4_ (0.1%), and MgSO_4_.7H_2_O (0.02%)). The fermentation broth was filtered under reduced pressure to afford the filtrate and mycelia. The filtrate was extracted three times with EtOAc, then the extraction was concentrated under reduced pressure to give a crude extract. The extract was subjected to column chromatography over silica gel eluted with a gradient of CHCl_3_/MeOH to obtain 2 fractions (1 and 2). Fraction 1 was loaded onto Sephadex LH-20 column and eluted with CH_2_Cl_2_-MeOH (1:1) led to Fraction 1a-1c. Fraction 1b was subjected to silica gel chromatography using PE/EtOAc mixtures to afford Fraction 1b1-1b3. Fraction 1b2 was purified by semi-preparative HPLC to afford atranone T and U (Qin et al. 2020).

### Atranones V-Z and Stachatranone D-F

The *S. chartarum* strain MK517540 was cultured on potato dextrose agar plates at 28 °C for 7 days to prepare the seed cultures. Agar plugs were then cut into small pieces and with 250 g of rice and 250ml of distilled water incubated at 28 °C. After 30 days, fungal growth was stopped by adding 95% EtOH to each flask, and the culture was homogenized. The suspension was extracted repeatedly with ethyl acetate for three times, and the organic solvent was evaporated under reduced pressure to yield a crude extract. The crude extract was fractionated on RP-C_18_ silica gel column chromatography eluted with MeOH–H_2_O to give four main fractions (A–D).

Fraction A was subjected to silica gel column chromatography eluted with PE-EtOAc to give four main fractions (A1–A4). Fraction A2 was further subjected to silica gel column chromatography, Sephadex LH-20 and semi-preparative HPLC to obain stachatranone D. Fraction A3 was separated by silica gel column chromatography and semi-preparative HPLC to afford atranone W, Y, and stachatranone F. Fraction A4 was repeatedly separated via semi-preparative HPLC to afford atranone Z, stachatranone E. Fraction B was loaded onto Sephadex LH-20 and silica gel column chromatography eluted with PE–EtOAc to give seven main fractions (B1–B7). Fraction B1 was separated via silica gel column chromatography, RP-C18 silica gel column chromatography, and semi-preparative HPLC to afford atranone V and X (Lin et al. 2024a).

### Methylatranone A and C

A mixture of atranones A and C was stored as a concentrated solution in MeOH at −5 °C for 2 months. TLC analysis revealed the presence of additional compounds, and the mixture was separated by radial chromatography to yield methylatranone A, methylatranone C and a mixture of atranone A and C (Hinkley et al. 2003).

### Methylatranone B and Benzylatranone B

Atranone B was dissolved in MeOH and formic acid added. The reaction mixture was stirred and heated at 60 °C in a sealed vial for 5 h. The reagents were removed by rotary evaporation, and the product was applied to a 1 mm silica chromatotron plate to yield methylatranone B. The benzyl derivative benzylatranone B was synthesized in a similar fashion using benzyl alcohol and TFA (Hinkley et al. 2003).

## Data Availability

No datasets were generated or analysed during the current study.

## Abbreviations

*ac1*: Atranone cluster 1
*ac2*: Atranone cluster 2
BALF: Bronchoalveolar lavage fluid
*cac*: Core atranone cluster
*ctc*: Core trichothecene cluster
EtOAc: Ethyl acetate
FFP: Farnesyl pyrophosphate
GGPP: Geranylgeranyl pyrophosphate
HPLC: High-performance liquid chromatography
IL: Interleukin
MeOH: Methanol
MIP-2: Macrophage inflammatory protein 2
PEI: Polyethyleneimine
PSDs: Phenylspirodrimanes
*S.*: *Stachybotrys*
TNF-α: Tumor necrosis factor-alpha
